# Testing for *Mycoplasma genitalium* in pelvic inflammatory disease: A clinical audit

**DOI:** 10.1111/ajo.13609

**Published:** 2022-09-11

**Authors:** Victoria Beesley, Caroline Thng

**Affiliations:** ^1^ Rotational Resident Medical Officer Gold Coast University Hospital Gold Coast Queensland Australia; ^2^ Sexual Health Consultant Gold Coast Sexual Health Service Gold Coast Queensland Australia

**Keywords:** Clinical audit, Macrolide resistance, Mycoplasma Genitalium, Pelvic inflammatory disease, Sexually transmitted diseases

## Abstract

The records of women attending Gold Coast health hospital sites were retrospectively analysed to determine if women diagnosed with pelvic inflammatory disease (PID) were being tested for *Mycoplasma genitalium* (MG). Only 11.4% of 299 women were tested for MG despite 74.2% being tested for *Chlamydia trichomonas* (CT) and *Neisseria gonorrhoeae* (NG). Only 9% of the women were treated with antibiotics which would treat macrolide‐sensitive MG infection. Increasing education and awareness of MG and utilising reflex macrolide testing for MG will help direct effective antibiotic therapy and prevent the long‐term sequalae of PID.

## INTRODUCTION

Pelvic inflammatory disease (PID) is a syndrome comprising of a spectrum of inflammatory disorders of the upper female genital tract, including any combination of endometritis, salpingitis, tubo‐ovarian abscess and pelvic peritonitis.^1^ The diagnosis of PID is challenging, as symptoms and signs lack sensitivity and specificity compared to diagnostic laparoscopy.^2^, ^3^ Conversely, invasive laparoscopy may be difficult to justify in patients with mild to moderate symptoms. Imaging techniques with either ultrasound, magnetic resonance imaging or computed tomography scanning can be helpful to differentiate PID from other alternative diagnoses.^4^, ^5^, ^6^

The long‐term sequalae of PID includes ectopic pregnancy, tubal factor infertility and chronic pelvic pain.^7^ Hence prompt diagnosis and appropriate treatment is important, and a low threshold of clinical suspicion must be exercised.^8^

Although up to 70% of cases have an unidentified cause, sexually transmitted infections (STIs) including *Chlamydia trichomonas* (CT), *Neisseria gonorrhoeae* (NG) and *Mycoplasma genitalium* (MG) have been implicated.^9^, ^10^ Guidelines for the management of PID recommend endocervical collection for culture and nucleic acid amplification test for STIs including CT and NG. More recently, following the widespread availability of MG molecular diagnostic tools, testing for MG has also been included in PID guidelines.^1^, ^11^, ^12^, ^13^

Investigating for MG infection is important as MG is not always adequately treated with principal PID antibiotic regimens, leading to persistent infection in patients with MG‐associated PID.^14^ The British Association for Sexual Health and HIV (BASHH) recommend a target of 90% of all women with suspected PID tested for MG.^12^ In Australia, there are no auditable standards for the testing of MG in patients presenting with PID.

The primary aim of the project was to establish if patients diagnosed with PID are being tested for MG as per Therapeutic Guidelines (eTG)^13^ and the Australian STI guidelines.^1^

## MATERIALS AND METHODS

A clinical audit was performed of patients presenting to Gold Coast health hospital sites from January 2019 to January 2020.

All data was extracted from the integrated electronic medical records (iEMR) which contains all clinical data including investigations and medications.

A list of eligible patients was generated from a report of all inpatient and outpatient presentations given a coded diagnosis which satisfied the spectrum of PID (Appendix A). The assigned codes were generated from treating clinicians' primary and secondary diagnoses at time of discharge.

Individual patients' electronic records were then reviewed to confirm inclusion based on clinical presentation, examination or laparoscopic findings which were consistent with PID. The Australian STI guidelines were used to benchmark the clinical definition for PID.^1^ Patients were excluded from the study if there was insufficient information to clearly define a diagnosis of PID (eg missing documentation), if they were treated elsewhere or if they re‐presented with the same symptoms within a 2‐week period (Appendix B).

The data collected included patient age at diagnosis, CT, NG and MG testing, and antibiotic therapy given as an inpatient, outpatient or on discharge. Descriptive statistical analysis was performed.

The study was approved by the Gold Coast hospital and health service human research ethics committee (EX/2021/QGC/81850).

## RESULTS

### Findings and discussion

Our findings are summarised in Table 1. There were 402 patients' notes including diagnoses which could indicate PID (Appendix A) which were reviewed; of these 299 were included for final analysis. Of patients included in the data set, 288/299 (96.3%) were of reproductive age (14–49 years old). Hence the implications of correctly identifying STIs as a curable causative agent for PID is important, to reduce significant reproductive morbidity.

**Table 1:** Demographics, treating department, testing and treatment received for patients with pelvic inflammatory disease

Patient demographics	*n*	%
Total patients	299	
Presentation		
Emergency department	128	42.8
Inpatient/gynaecology	171	57.2
Age		
<15	1	0.33
15–19	32	10.7
20–29	125	41.8
30–39	79	26.4
40–49	52	17.4
>49	10	3.3
Testing		
Tested for CT and NG by PCR	220	73.6
Tested for CT only	2	0.6
Tested for MG by PCR	34	11.4
Treatment		
Effective MG treatment given	27	
Yes	227	9.0
No	45	75.9
Missing data		15.1

Abbreviations: CT, *Chlamydia trichomonas*; MG, *Mycoplasma genitalium*; NG, *Nisseria gonorrhoeae*; PCR, *polymerase chain reaction*.

There were 222/299 (74.2%) patients diagnosed with PID tested for CT or NG, but only 34/299 (11.4%) of patients were tested for MG. This highlights an awareness of testing for STIs, but a missed opportunity to appropriately include MG testing in patients with PID. All patients who were tested for MG had CT and NG tests.

In our study, only 9% of patients treated for PID received antibiotics which would have treated macrolide‐sensitive MG infection.

Our study demonstrates that although healthcare professionals are aware of the need to test for STIs (CT and NG) in PID they are less aware of the need to request MG tests. It suggests that education and a review of guidelines used in our emergency and gynaecology departments is required to increase MG testing rates for patients diagnosed with PID.

Diagnosing MG‐associated PID is important because: (1) empirical principal antibiotic regimens for PID do not adequately treat MG infections, potentially leading to long‐term sequelae;^1^, ^4^ (2) MG antibiotic resistance (to both macrolides and quinolones) is an emerging concern with studies suggesting macrolide‐resistant MG infections in Queensland as high as 60%, and quinolone resistance at 10%;^15^, ^16^, ^17^ (3) reflex testing for macrolide resistance is now widely available to facilitate correct antibiotic prescribing and prevent further emergent resistant MG.

There are several limitations to our study. The diagnosis of PID includes a spectrum of presentations and hence it is difficult to determine the completeness of our patient list. However, all notes that were included were reviewed to ensure that patients who were included fitted a clinical diagnosis of PID as per the Australian STI guidelines. Also, our study is limited to a single site, and hence larger studies would be required to determine the scale and potential impact of undertested MG in patients with PID. Further qualitative studies would be required to determine the barriers and reasons for low MG testing rates and to inform intervention strategies to improve testing rates for MG in patients diagnosed with PID.

## CONCLUSIONS

Testing for MG in patients diagnosed with PID remains low despite recommendations in current guidelines. This is a missed opportunity for prompt diagnosis and effective treatment, not only for positive patient outcomes but also to reduce the cost of managing the long‐term sequalae of PID.^18^ Further research into the determinants of MG testing among patients diagnosed with PID by clinicians may help guide interventions to improve MG testing.

The current prevalence of MG, which is relatively low,^19^, ^20^ balanced with the potential toxicities of antibiotics required to adequately treat MG, does not justify changing guidelines for the empirical treatment of PID. Instead, we can capitalise on current high testing rates for CT and NG in patients with PID to include MG testing and the availability of reflex macrolide testing for MG to ensure adequate and appropriate treatments to improve patient outcomes.

## Appendix A

### A.1 A table showing the coded diagnosis of patients included in the final data collection set

**Table:**

Coded diagnosis
Acute salpingitis and oophoritis
Salpingitis and oophoritis unspecified
Acute inflammatory disease of uterus
Inflammatory disease of uterus unspecified
Inflammatory disease of cervix
Acute parametritis and pelvic cellulitis
Female acute pelvic peritonitis
Female pelvic peritonitis unspecified
Female pelvic peritoneal adhesions
Other specified female pelvic inflammatory disease
Female pelvic inflammatory disease unspecified
Female gonococcal pelvic infectious disease
Female chlamydial pelvic infectious disease
Female pelvic infection disorder in other disease
Other specified inflammation of vagina and vulva
Endometritis
Acute endometritis
Infection of uterus

## Appendix B

### B.1 A table showing the inclusion and exclusion criteria of the clinical audit

**Table:**

Inclusion criteria	Exclusion criteria
Diagnosis consistent with PID including cervicitis, endometritis, salpingitis, tubo‐ovarian abscess and pelvic peritonitis	Patients representing within 2 weeks of initial diagnosis
Diagnosis of PID made from presentation consistent with signs and symptoms of PID^1^	No access to pathology testing performed
Diagnosis of PID made from positive endometrial or cervical sampling	Testing and diagnosis of PID made at external site
Diagnosis made from positive laparoscopy findings	
Diagnosed and treated between 01/01/2019–01/01/2020	

Abbreviation: PID, pelvic inflammatory disease.
